# Some Observations on Surgical Dressings and the Care of Wounds

**Published:** 1907-10

**Authors:** J. H. Johns

**Affiliations:** Demonstrator of Anatomy and Assistant to Chair of Clinical Surgery, College of Physicians and Surgeons, Atlanta, Ga.


### SOME OBSERVATIONS ON SURGICAL DRESSINGS AND THE CARE OF WOUNDS.

By J. H. Johns, M. D.
Demonstrator of Anatomy and Assistant to Chair of Clinical Surgery, College of Physicians and Surgeons, Atlanta, Ga.

The frequency with which I see wounds, especially in clinical work, where the physician giving first attention has used a dry dressing with some powder, usually Iodoform, and my belief in the superiority of the moist dressing, the reason for which I shall endeavor to point out, are my excuses for offering a short paper on this subject.

We all realize, or should do so, the importance of sufficient drainage. Even in operative wounds where we have every reason to believe everything is clean, we have more or less serous oozing from the wounded tissue for the first while. A moist dressing absorbs this serum much more readily than a dry dressing and has not the same tendency to stick to the wound, consequently is much more comfortable for the patient. (In these cases a moist saline or very mild antiseptic dressing is preferable.) Where we have a wound in which we have reason to expect infection, a moist dressing is much more important.

I have often seen patients with a contused or lacerated wound which had been tightly sutured and dressed with Iodoform powder and dry gause or with Collodion, with the result that the serum which oozed from the wounded tissues, being unable to get out through the practically waterproof dressing, had accumulated in the wound; stretching apart the surfaces which should come in contact in order to heal, causing so much pain from pressure that in many cases Morphine had been given to relieve it, and furnishing a most admirable culture medium for any pyogenic organisms which may have escaped destruction in the cleansing process. A moist antiseptic dressing of either weak Bichloride or one of the many antiseptic solutions owing their effect to Menthol, Thymol, Boric acid, etc., after removing the crusts formed by the powder, will do more to relieve the pain in these cases than an opiate; and if such a dressing be used in the beginning the patient will not only be saved a considerable amount of discomfort, but the liability to infection will be reduced probably seventy-five per cent. and many limbs saved which will otherwise be amputated.

In this connection will say that the smallest number of sutures that will approximate the wound edges well should be used, and these tied just tightly enough to bring the edges lightly together. All we need is contact of the raw surface. Forcibly drawing the edges together does not promote healing, on the contrary, it retards healing by strangulating the tissues within the grasp of the suture.

As a primary dressing, a moist dressing is superior to a dry dressing or Collodion in all cases. In clean cases after the period of serous oozing (24 to 36 hours) a dry dressing is preferable as it has a tendency to render the skin harder and the stitches will not cut so badly. This is especially to be remembered when there is considerable tension on the stitches. In infected wounds when it has been necessary to use a moist dressing for some time before the infection is controlled, we find the tissues about the wound have puffy or "soggy" appearance, then a dry dressing with some dusting powder is indicated.

As a dusting powder in surgical dressings, for general use, I prefer Crystals of Acetanilid. It does not form a hard scab and is non-irritating. Iodoform has its field to stimulate granulations, Compound Alum powder for excessive granulations, etc.

For superficial abscesses, such as Bubo, Suppurating Tubercular Glands, Boils, etc., I have, for several years, used Carbolic Acid and Alcohol. After evacuating the pus, the abscess cavity is mopped out with a swab of cotton twisted on an applicator and dipped in pure liquid Carbolic Acid, first surrounding the opening with a piece of absorbent cotton saturated with Alcohol, and followed immediately with a mop saturated with Alcohol full strength. The cavity is then packed with a strip of gauze wrung out of 1 to 2000 bnchloride and saturated with Alcohol which is allowed to remain twenty-four to thirty-six hours. When this packing is removed it will bring with it, if the first dressing has been thorough, all the so-called Pyogenic lining of the cavity, leaving a clean surface which is ready to granulate. At the second dressing, if the cavity is reasonably superficial, I simply pack it with Crystals of Acetanilid and put on a gauze compress.

Another useful dressing to which I wish to call the attention of those who have not used it, is Ichthyol Ointment 25 per cent. This is one of the very best dressings, especially in inflammations of the phlegmonous type such as we often see in the palm. After free incision, the ointment should be applied freely to the wounds as well as to the whole skin area where there is swelling, redness, or tenderness. A very thin layer of gauze should be applied next to the ointment and over this cotton batting. The dressing should be changed every 12 to 24 hours until the infection is controlled.

I had a patient some time ago who was well and at work, as a sheet metal worker, two weeks after I first saw him with an extensive palmar abscess which I treated in this way. I have used this dressing in many such cases as well as in bone felons with equally good results. Ichthyol is a common application for Erysipelas and subcutaneous infections, but I don't think it is used as much in infested wounds as its efficiency would indicate.

I wish before closing to protest against too much irrigation and packing, as well as too frequent dressing. We can keep a wound open almost indefiniately by daily irrigation and packing.

We often don't seem to realize that it is worse than a waste of time to change a clean dressing on a clean wound, especially on a granulating wound, where more or less of the granulations are destroyed at each dressing and the healing process just that much delayed. This is especially true where such a wound is flooded with strong Bichloride or some other antiseptic at each dressing.

The three principal reasons for changing a dressing are: When the dressing is soiled, when we have an indication for inspection of the wound, or when we wish to make some application to the wound.

Dr. John B. Deaver's motto for his house staff: "Let the patient get well," is applicable to dressings in many cases.

We must not lose sight of the fact that the care of the wound is in many cases as important as the operative procedure and in some cases more so. A dressing should be used which is indicated by the condition of the wound at that time, and not the routine application of some dressing which is commonly used.

